# Contribution to the Causistry of Hereditary Syphilis

**Published:** 1882-01

**Authors:** W. Lewin, P. W. Van Peyma

**Affiliations:** Friedrichsberg


### Translations.

---

### Contribution to the Causistry of Hereditary Syphilis, by Dr. W. Lewin, of Friedrichsberg.

From the German by P. W. Van Peyma, M. D.

In No. 3 of the *Berliner Klinische Wochenschrift*, 1876, Prof. Lewin in his article upon hereditary syphilis, calls attention to a very characteristic symptom of this disease, the separation of the epiphysis from the diaphysis of the tibia. Since the number of cases in which this condition was recognized during life is still limited, I take the liberty of reporting the two following cases. The first of these, I observed in January, 1876. On the 20th of the month mentioned, I was called to see a child four months of age. As I entered the room, a noisy snuffling of the child attracted my attention. There existed a purulent discharge from the nose, with erosion of the nostrils and the upper lips, and rhagades at the corners of the mouth. The eyes were inflamed, the forehead glistening, indicated a former eruption. The skin was generally dry and scaly. Both arms were enclosed at their lower third in cotton batting. A physician previously called having declared both arms to be fractured, and in truth an exquisite crepitation was obtained upon moving the epiphyses upon the diaphyses, proving that the former had become separated from the latter.

That this condition was a result of congenital syphilis admitted of no doubt. The subsequent anti-syphilitic treatment failed to ward off death, the child dying ten days after from exhaustion, having been unable to nurse on account of the rhagades about the mouth.

The second case I observed during December of the preceding year. At eight in the morning I was called to the house of coachman L., of Berlin, whose child had been ill for a considerable time. I found the same to be four months old, and extremely cachetic. The skin was dry and scaly. The extremities presented a limited scaly eruption. The nostrils as well as the upper lip were eroded, the conjunction swollen so that it was with difficulty that the orbit could be uncovered. The palms of the hands and soles of the feet were characteristically glistening, as I have often observed after the healing of syphilitic eruptions. For these reasons I was convinced that the case was one of congenital syphilis. This conviction was strengthened to absolute certainty when the mother of the child informed me of the fact that the left arm as well as the right knee presented a tumefaction. Upon rubbing the epiphysis of the left humerus against the diaphysis of the same, a distinct crepitus was felt. A similar result was obtained upon moving the lower epiphysis of the right femur against the diaphysis of this bone. Both epiphyses were also decidedly enlarged. By percussion I could not determine that the liver and spleen were enlarged.

Upon remarking to the mother that the child must have suffered from an eruption some weeks after birth, she replied that the child had been covered with red spots which later had desquamated. The child died after twenty-four hours.

The first of the cases reported, presented some additional points of interest. The mother of this child lived in concubinage with a man in whom, upon examination, I was unable to find any indications of syphilis. An examination of the mother gave likewise negative results. She, however, informed me that she had previously had a child by another man, which child, according to the attending physician, had died of enlargement of the liver and spleen. With good reason it may be assumed that this child was syphilitic. If this was the case, it furnishes a confirmation of the views first advanced by Furth and Caspary that "women who bear syphilitic children by syphilitic fathers, are themselves infected, even though they present no apparent symptoms of syphilis." For the father of this child was without doubt perfectly healthy.

A similar observation that I had opportunity to make sometime since confirms this view.

On the twenty-eighth of December, 1874, I was called by B., by occupation a potter, to see a seven months' fœtus which had died one-half hour after delivery. It presented a well-developed syphilitic pemphigus of the palms of the hands and soles of the feet. Upon closer inquiry I learned that the mother had previously aborted at three months. Neither she nor her husband, who willingly submitted himself to examination, presented symptoms of a former infection. The woman, however, informed me that she was married for the second time, and that her first husband died of syphilis in a Hamburg hospital.

Here also the wife of a syphilitic husband remained apparently healthy, notwithstanding which she afterward gave birth to a syphilitic child by a healthy husband.

